# Framing analysis as a pedagogical tool for broadening medical students’ understanding of cancer: a course evaluation

**DOI:** 10.1186/s12909-026-09851-y

**Published:** 2026-07-03

**Authors:** Jarle Breivik, Hanne Cathrine Lie

**Affiliations:** Department of Behavioural Medicine, Institute of Basic Medical Sciences, University of Oslo, P.O. Box 1110 Blindern, Oslo, 0317 Norway

**Keywords:** Medical education, Cancer communication, Framing analysis, Interdisciplinary learning, Critical thinking, Reflective practice, Media literacy

## Abstract

**Background:**

Cancer is a complex phenomenon encompassing a range of cultural and biomedical dimensions. Traditional medical curricula often fail to reflect this complexity, highlighting the need for interdisciplinary approaches that help medical students develop a more integrated understanding of disease by connecting diverse knowledge, patient perspectives, and societal contexts. This study evaluates how framing analysis—a method from communication studies—can be used as a pedagogical tool to broaden medical students’ understanding and critical thinking about complex health issues, using cancer as a key example.

**Methods:**

We conducted a course evaluation of a two-week elective titled “Cancer Communication” within the University of Oslo’s medical program, offered annually to approximately 20 third-year students. Working in teams, students explored research questions related to cancer communication through lectures, workshops, guided research, and excursions. They learned framing analysis by applying a published tool for examining public cancer discourse. Anonymous pre-course free associations and post-course reflections were analyzed thematically to identify changes in students’ understanding of cancer during the course. Students’ team presentations, orally evaluated as part of the final course examination, supported our assessment of how students applied framing analysis to cancer-related communication topics.

**Results:**

Students’ initial responses reflected relatively simple and emotionally charged associations with cancer, often centered on fear, death, treatment burden, and familiar cancer stereotypes. Their post-course reflections suggested increased media literacy, critical thinking, and the ability to integrate multiple narratives about cancer. In their final presentations, students applied framing analysis to critically evaluate media portrayals and reflect on how different framings shape public and professional perceptions of disease. The post-course reflections further suggested greater empathy for cancer patients and an increased awareness of social and cultural influences on medical practice.

**Conclusions:**

Integrating framing analysis into medical education appeared to broaden students’ understanding of cancer as a multidimensional phenomenon, supporting reflective and interdisciplinary thinking and encouraging them to move beyond traditional biomedical perspectives. These findings suggest that framing analysis and similar communication-based approaches may serve as useful pedagogical tools in health professions education to enhance critical reflection.

**Supplementary Information:**

The online version contains supplementary material available at https://doi.org/10.1186/s12909-026-09851-y.

## Background

Cancer is not only a leading cause of morbidity and mortality worldwide [1]; it embodies a complex interplay of biomedical, psychosocial, cultural, ethical, economic, environmental, and existential dimensions [2]. Narratives surrounding cancer are often diverse and sometimes conflicting, ranging from aggressive war metaphors and promises of the ultimate “cure” to the sobering realities of epidemiology and personal suffering [3]. Developing the ability to navigate this complexity represents an essential goal for future physicians and poses an ongoing challenge for medical training.

Traditional biomedically oriented curricula often struggle to provide the insights and critical perspectives necessary for meaningful engagement with the multifaceted nature of cancer-related information [4, 5]. Given this deficiency in educational approaches, there is a need for innovative learning designs that adopt interdisciplinary and integrative approaches to cancer education, promoting critical thinking and facilitating a deeper understanding of the underlying issues [6].

Taking a constructivist perspective on education [7], we argue that medical students need to engage with the phenomenon of cancer from multiple angles, utilizing a range of sources and experiences [8]. Learning is an intrinsically active, contextual, and social process [9]. To fully grasp the complexities of cancer, and other diseases, medical curricula should foster learning environments where students interact, collaborate, explore, and reflect, thereby creating meaning through experience and dialogue [10]. They should be encouraged to question assumptions, embrace uncertainties, and synthesize diverse viewpoints [11]. Such engagement may support deeper, reflective learning [12], helping students develop integrated models of understanding that make sense of both cancer and the broader complexities of medicine.

In the social sciences and communication studies, such sense-making and the creation of meaning are often associated with the concept of framing. Erving Goffman described framing as the cognitive structures individuals use to organize and interpret their experiences [13], while Robert M. Entman emphasized how news media employ framing to shape public understanding by highlighting certain aspects of information [14]. Framing analysis thus provides a structured approach to examining how language, metaphors, and narratives influence perception. As a qualitative research method, framing analysis may be applied inductively, to identify emerging patterns and themes, or deductively, to apply predefined schemes to new materials [15].

Carver et al. demonstrated how framing analysis can enhance science education by changing how students interpret complex genetic concepts [16]. Similarly, Murray et al. developed a framing scheme for analyzing public cancer discourse across nine distinctive perspectives [2].

Building on this work, the present study evaluates the use of “The Nine Cancer Frames” as a pedagogical tool in a two-week elective course on cancer communication. The course was designed to broaden students’ understanding of cancer as a complex biomedical, cultural, and social phenomenon while supporting critical engagement with cancer-related discourse. Specifically, we asked: (1) How did students engage with and apply framing analysis in the context of cancer communication? (2) How did students’ pre-course associations and post-course reflections suggest changes in their understanding of cancer and its broader meanings?

## Materials and methods

### Course design and implementation

In 2017, the six-year medical program at the University of Oslo introduced a portfolio of two-week elective courses as part of the third-year curriculum [17]. These courses followed the introductory clinical skills and examination component and provided opportunities for in-depth exploration of topics beyond the standard curriculum. The course titled “Cancer Communication” was designed to broaden students’ understanding of cancer while promoting critical thinking and communication skills. As of 2025, the course had been offered for nine consecutive years, enrolling approximately 20 students each year. The present study was designed as a course evaluation based on anonymized educational data collected from the 2023, 2024, and 2025 course iterations.

Students ranked six of the eleven available electives and were enrolled based on course capacity. At the cohort level, the course was ranked as first choice by 27 of 201 students in 2023, 36 of 254 in 2024, and 35 of 245 in 2025, exceeding the approximately 20 available places each year and suggesting substantial student interest. Of the 60 students who participated across the three course iterations included in this evaluation, only four (7%) were male. Given that approximately 30% of the total student population was male, this distribution indicated a marked gender imbalance in course enrollment. No other demographic data were collected.

Besides adaptations for online and hybrid education during the COVID-19 pandemic and minor adjustments for quality improvement, the basic design remained consistent over the nine-year period: The students were randomly divided into four teams of five. The first day focused on teambuilding activities to establish psychological safety and reach mutual agreement on collaboration. Each team was then assigned a unique research question related to different aspects of cancer communication. New research questions were introduced each year, with typical examples including: “How is dying from cancer framed in popular culture, and what can we learn from this?” “What does it mean to be a cancer survivor, and how is this framed in the media?” and “How is cancer research presented in the news, and how do the narratives correspond to scientific reality?”

The first week focused on information gathering, during which the students participated in lectures and workshops that presented different perspectives and discourses about cancer. This included meetings with cancer patients and oncologists, as well as excursions to the Norwegian Cancer Society, the Cancer Registry of Norway, the Norwegian Radium Hospital, and the Munch Museum. The session at the Munch Museum was specifically designed to explore themes of death through the paintings of Edvard Munch, facilitated by an art historian and a palliative care physician [18].

During the second week, the students worked in teams to systematically collect and analyze data related to their research questions, while the course leaders (the authors) provided supervision throughout the process. The students received a framework for presenting their research based on the IMRAD structure [19], along with explicit criteria for evaluation (Supplement 1).

For each presentation, one peer group was assigned to lead the discussion and provide feedback on the work using the predefined criteria. This was followed by a formative discussion involving peers, faculty, and representatives from the collaborating institutions. The course leaders then made the final summative pass/fail assessment. To date, no student group has failed the final examination; however, individual students with insufficient participation have been required to complete additional written assignments to pass the course.

### Framing analysis

The course employed framing analysis in three ways: as an educational tool for teaching critical thinking, as a research method for the students to analyze their material, and as a skill and learning outcome that the students would develop throughout the course. The pedagogical sequence was designed to move students from conceptual introduction, to guided deductive application of an established framing scheme, and finally to independent use of framing analysis in their team projects.

On the second day, the students were introduced to the concepts of framing and framing analysis through an interactive workshop, including two educational YouTube videos presenting the concept of framing from distinctly different perspectives [20, 21]. This approach offered broad insights and illustrated how even the concept of framing itself can be framed in different ways, allowing the students to engage critically with the material.

**Fig. 1:**
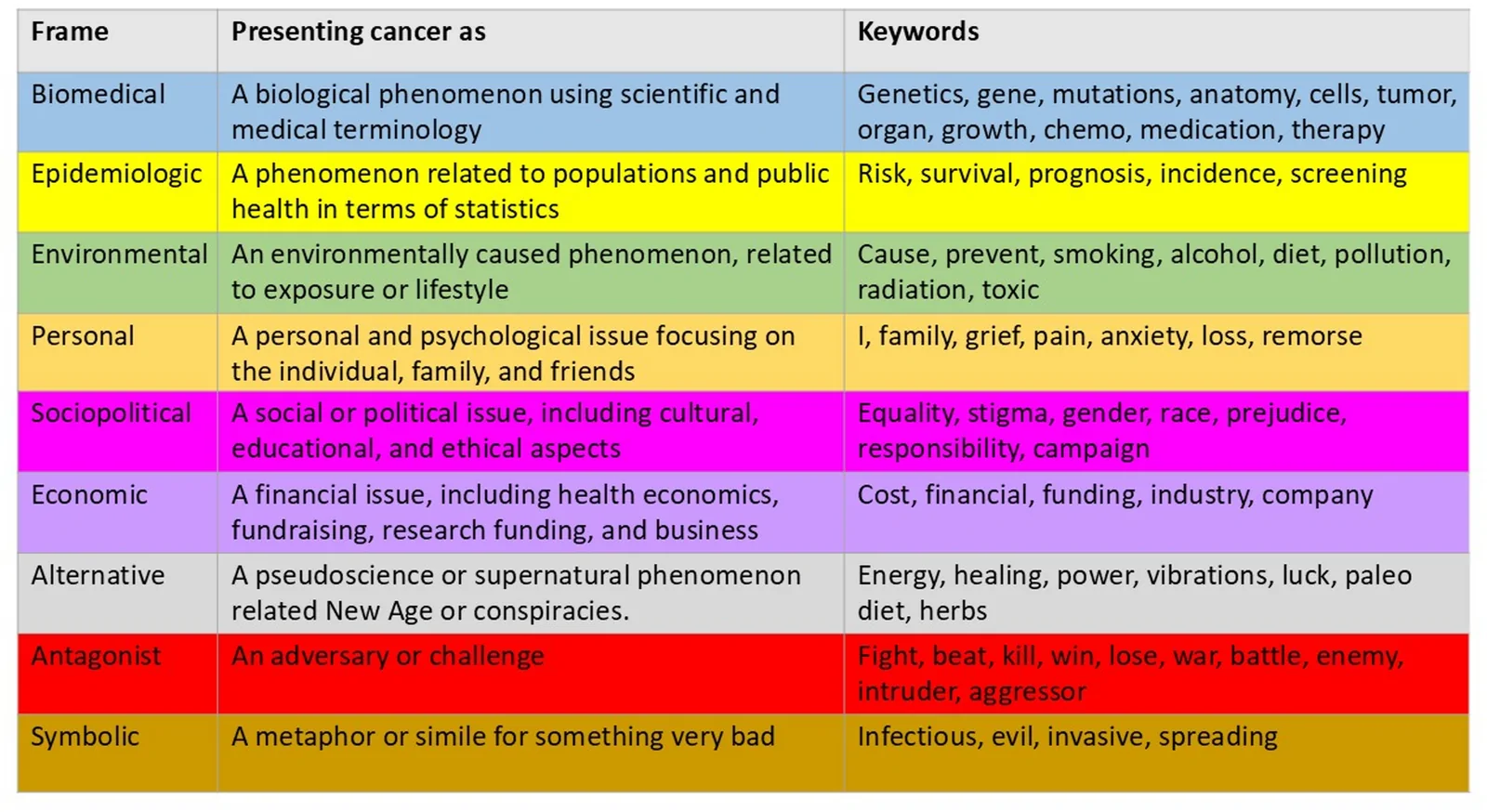
The cancer framing scheme developed by Murray et al. [2] was provided to students as a handout, which assigned a distinct color to each of the nine frames. The students were also given marker pens in corresponding colors, which they used to identify relevant text in cancer-related news articles as well as their own free associations about cancer (the pre-test)

They were then assigned to read “The Nine Cancer Frames: A Tool to Facilitate Critical Reading of Cancer-Related Information” by Murray et al. [2]. The article presents a deductive framing scheme consisting of nine cancer frames: the biomedical, epidemiological, environmental, personal, sociopolitical, economic, alternative, antagonistic, and symbolic cancer frames. This scheme was provided to students as a color-coded handout to support systematic analysis of cancer-related material (Fig. 1).

**Fig. 2:**
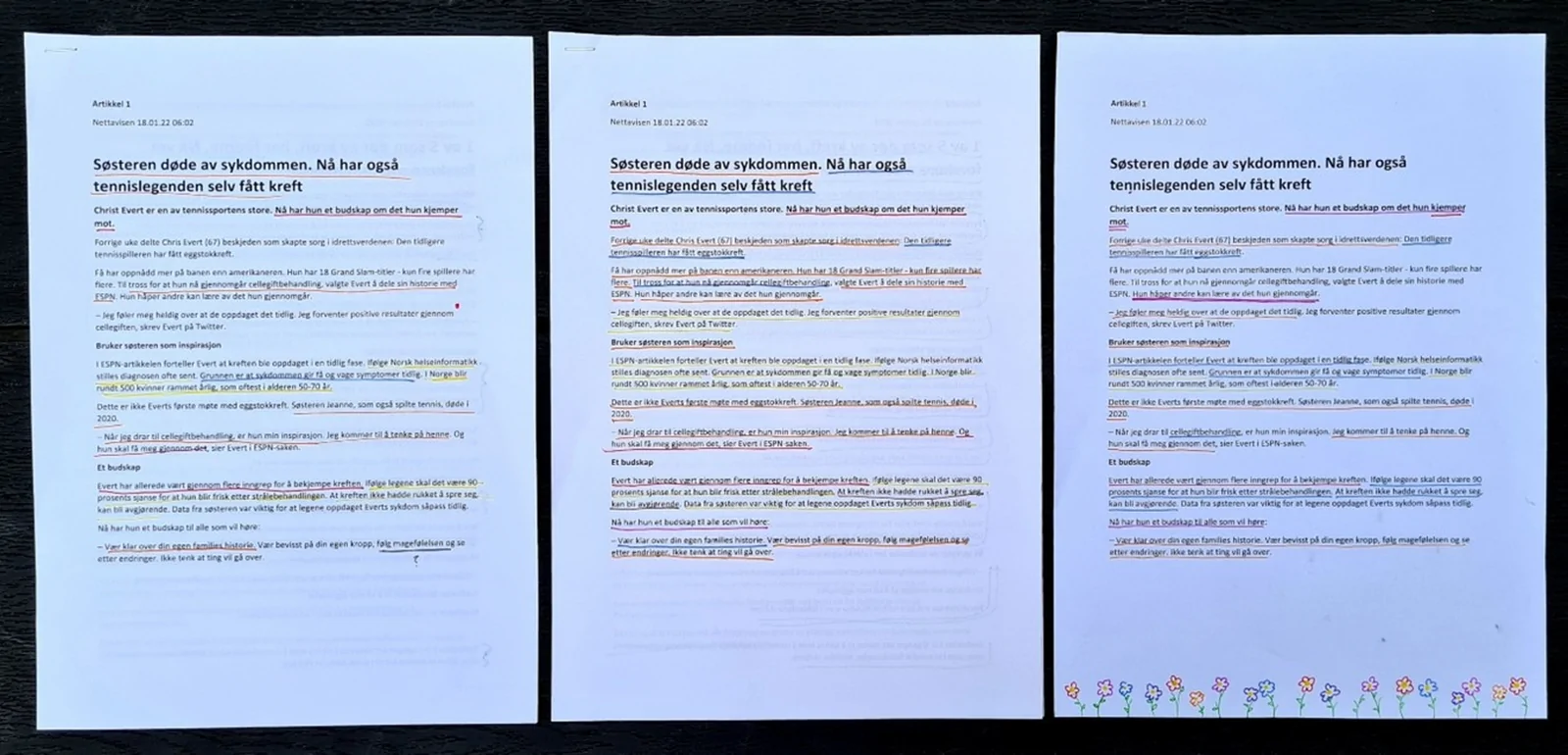
Coding of a newspaper article by three independent students based on the framing scheme developed by Murray et al. [2]. This selection illustrates both the general agreement and the differences in the students’ interpretations, establishing a basis for fruitful discussion about framing analysis, medical communication, and different perspectives on cancer communication

Using the color-coded framing scheme, the students identified which cancer frames were present in two separate news articles, highlighting relevant passages with correspondingly colored markers (Fig. 2). They then compared and discussed their analyses with their team members before sharing their findings and experiences in a plenary session. This comparison was intended to help students move beyond identifying individual frames towards interpreting how different framings shape meaning, emphasis, and possible reader responses. Subsequently, the students applied the same scheme to analyze their own pre-collected free associations about cancer (see Course Assessment) and were encouraged to reflect on their collective preconceptions.

Finally, the students considered how framing analysis could be utilized to explore their assigned research questions, either by inductively identifying novel frames related to their specific topic or by deductively applying Murray et al.’s [2] framing scheme to analyze their data. Through this process, the students were challenged to apply framing analysis to answer their own research questions, asking not only which frames were present, but also what those frames emphasized, obscured, and implied for the understanding of cancer.

### Data collection and assessment

At the start of the course, the students engaged in an anonymous pre-test activity aimed at capturing their initial assumptions and attitudes toward cancer. This involved a seven-minute exercise in which they anonymously shared their free associations with the word “cancer” in an online form, using single words, phrases, or complete sentences. Across the three course iterations, 52 of 60 students (87%) completed the pre-test activity. This aggregated response provided a baseline assessment of the students’ perceptions of cancer, which they would later revisit for analysis and reflection.

Upon completing the course, the students were invited to participate in an anonymous online survey designed to evaluate the course’s effectiveness in achieving its aims of broadening students’ understanding and enhancing their critical thinking about cancer-related communication. Norwegian and English versions of the questionnaire are provided in Supplement 2. The survey included the following items: “What is the most important thing you learned in this course?” “Explain how the course has influenced your view of cancer?” and “How do you think what you have learned might influence you in your further studies and medical practice?” These end-of-course reflections also served as a post-test, complementing the pre-test. Across the three course iterations, 50 of 60 students (83%) completed the survey.

From a methodological standpoint, using identical questionnaires for the pre-test and post-test may seem like a more stringent approach. However, anticipating that students’ perspectives and context would be heavily influenced by the course, including the questions we asked, we concluded that comparing their initial, naive free associations with cancer to more directed reflections at the end would provide a more insightful assessment of the learning process. As the two data sources differed in format, comparisons were interpreted as qualitative changes in students’ expressed understanding rather than direct measurements of learning outcomes. This design was thus intended to support a qualitative course evaluation rather than a controlled pre-post measurement of learning outcomes.

The results of both the pre-test and post-test were subjected to thematic analyses [22] to identify and categorize key themes and patterns within the students’ thoughts and reflections on cancer. First, the responses were thoroughly read to identify initial patterns, and significant phrases and ideas were coded across the dataset. Related codes were clustered to generate emerging themes that characterized the pre- and post-test results, respectively. To verify the reliability of the themes, two researchers (the authors) independently reviewed the codes and themes, discussing discrepancies to reach a consensus. Finally, the results from the two tests were compared to identify changes in the students’ understanding and perspectives on cancer. The course and assessments were conducted in Norwegian, and the presented quotes are translations. Quotes in Norwegian are available upon request from the authors. The students’ final team presentations were evaluated orally as part of the course examination and informed our assessment of students’ practical application of framing analysis but were not included in the thematic analysis.

All findings should be viewed in the context of potential bias, as the researchers also served as the course’s designers and leaders. Both authors were involved in developing, teaching, supervising, and evaluating the course, and both have academic interests in cancer communication, medical education, and reflective learning. This dual role may have shaped the design of the course, the interpretation of students’ responses, and the emphasis placed on positive educational outcomes. To ensure accountability and transparency, student surveys were shared unedited with the medical program administration. Additionally, the course was evaluated with standardized surveys for students and course leaders, provided by the administration, to assess quality and identify areas for improvement.

Data were collected anonymously as part of the University of Oslo’s educational quality-assurance procedures. The use of anonymized course-evaluation data is described in the Ethics approval and consent to participate section.

## Results

### Understanding and application of framing analysis

The students were able to apply the concept of deductive framing analysis during the initial workshop, using color coding to identify cancer frames in news articles. Overall, there was general agreement among the students and with the instructors, indicating that the framing scheme could be applied consistently in this educational setting. Some discrepancies were noted, and as emphasized by Murray et al., the boundaries between the nine cancer frames can sometimes be unclear. Rather than being treated as a problem, these ambiguities became a productive part of the learning process, prompting students to justify their interpretations, consider the boundaries between different cancer frames, and discuss how language, context, and perspective shape meaning in cancer communication.

By performing a framing analysis on their own free associations about cancer (the pre-test), the students identified a clear emphasis on the *biomedical*,* personal*, and *antagonistic* frames. The *environmental* and *economic* frames were scarce, while the *alternative* frame was absent. These observations prompted self-reflection and engaged the students in a deeper discussion about framing and their own preconceptions about cancer.

The team presentations provided evidence that students were able to independently apply framing analysis, in both an inductive and deductive manner. One team examining misinformation about cancer on social media identified three distinct frames: *advice*, *warnings*, and *conspiracies*. Another team, investigating the portrayal of artificial intelligence in cancer research, observed a predominantly *positivistic* frame that communicated unrealistic expectations and lacked critical perspectives. A third team analyzing gender differences in cancer communication found that narratives about male cancer patients often emphasized *performance*, typically linked to sports or sex, whereas stories concerning female patients were more frequently framed around *emotions* and *empowerment*. Some teams related their findings to established research literature, while others identified patterns that they considered relevant for further investigation.

The students’ feedback at the end of the course also suggested substantial engagement with framing and framing analysis. Asked about their key takeaways, many recognized the impact of framing on public perception, emphasizing the necessity of media literacy and the ability to critically evaluate information: One student wrote *“I learned to be more critical of how cancer is discussed in the media. Cancer can be framed in so many different ways*,* so you must be careful about what you trust.”* Several reflections indicated an understanding of how framing can influence emotions, decisions, and societal views, with one student notably expressing: *“The most important thing I learned from the course is that the way we communicate about cancer greatly affects our associations and perceptions of both cancer as a disease and all the other areas it touches.”* Another highlighted the importance of “*keeping framing analysis in the back of my mind”* when assessing different sources of information.

### Perspective on Cancer

The thematic analysis of the students’ free associations with cancer at the beginning of the course (the pre-test) revealed several salient themes (Table 1). One prevailing theme was *fear and uncertainty*, with words like “fear,” “death,” “pain,” “hopeless,” “scary,” “unfair,” and “sadness” frequently mentioned, highlighting the substantial emotional burden associated with cancer. Another theme, *negative impact on quality of life*, emerged through terms, such as “demanding treatment,” “fatigue,” “fragility,” “long-lasting,” and “bedridden,” indicating a widespread perception of cancer as a challenge affecting patients’ well-being and daily lives. These negative connotations were further emphasized by associations with negatively charged words related to cancer treatment like “chemotherapy,” “radiation,” “nausea,” and “hair loss,” reflecting an emphasis on the physical and psychological toll of cancer treatments. Additionally, the theme of *family and societal impact* was prominent, with mentions of “family,” “support,” and “loss,” underscoring the broader implications of a cancer diagnosis on social structures and relationships.

**Table 1:** Thematic analysis of pre-course associations and end-of-course reflections regarding the students’ understanding of cancer

Pre-course associations		
Theme	Description	Supporting Quotes
Fear and Uncertainty	Expressed through negative associations that emphasize the emotional burden of cancer.	“Scary.” “Inevitable?” “Uncertain future.” “Difficult message to give. Difficult message to receive.” “There is a lot of uncertainty afterwards (cancer-free, for how long?).”
Negative Impact on Quality of Life	Highlighted the physical and emotional challenges faced by patients.	“Reduction in quality of life.” “Losing hair, nausea, pain.” “Incurable.” “A lot of sorrow.” “Cancer treatment is a significant psychological and physical strain.”
Family and Societal Impact	Emphasized the broader implications of a cancer diagnosis on relationships and social structures.	“Cancer is hard to go through for the person diagnosed, but also hard for the relatives.” “Friends and acquaintances may distance themselves and dare not ask how the illness is progressing.” “Much talked about in the media.” “Norway is a country with a lot of cancer.”

**End-of-course reflections**		
Theme	Description	Supporting Quotes
Critical Engagement and Media Literacy	Students recognized the importance of discerning biases in media representations.	“I have learned to be critical and analyze information online.” “It is important to be critical of the sources regarding how the media presents cancer.” “Cancer can be framed in very many different ways, and it is important to be aware of and critical of what one reads in the media.”
Broadened Perspective	Students began to view cancer as a complex issue, acknowledging its multifaceted nature beyond mortality.	“Cancer is about much more than just death.” “Cancer is not one disease, but many.” “It has given me a somewhat different view of cancer patients and how they wish to be received and communicated with by us clinicians.” “It is a quite complex view, and that cancer is much more than just cancer.”
Communication and Empathy	Emphasized the significance of effective communication in patient care.	“I want to be more aware of the patients’ psychology and try to assure them that I want to help them with all aspects of cancer disease.” “How important it is to communicate about cancer, and how crucial it is to be careful with the words one uses.” “The most important thing I have learned in the course is that the way we communicate about cancer has a significant impact on our associations and perceptions.” “To talk about death with each individual patient.” “It is important to maintain good communication with patients, even though it can be challenging in certain situations.”
Viewing Cancer as Part of Life	Reflections included acceptance and understanding of cancer as a common aspect of human experience.	“Cancer is something one might not try to cure entirely, but rather a disease that many will get and live with.” “That it will never be possible to completely eliminate cancer from the world.” “It may not just be about the cancer itself, but to look for other solutions.” “I have become less afraid of cancer and now associate it more with a disease that one can often recover from.”

The thematic analysis of the students’ end-of-course reflections (the post-test) revealed a different picture (Table 1). Most obviously, the students exhibited *critical engagement*, advocating the importance of media literacy and skepticism towards how cancer is presented by the media. This was captured in reflections like “*Cancer is often portrayed as a battle in the media. This can make people feel like failures if they don’t ‘win’ the fight. We need to learn to think more critically about how cancer is talked about.”* Such reflections suggested that students were beginning to understand framing as more than a feature of communication, recognizing how language and metaphor can shape the meaning of cancer.

Another key theme was the *broadened perspective*, where the students recognized cancer’s complex nature beyond the biomedical frame and the war and fight metaphors. This insight was articulated through comments such as *“I have reflected on what it is like to live with cancer*,* how healthcare professionals interact with cancer patients*,* the weaknesses and strengths of current cancer care*,* existential questions related to being human*,* how cancer is communicated*,* and how the way we talk about cancer significantly affects how we cope with it both as patients*,* relatives*,* healthcare professionals*,* politicians*,* etc.”*

This quote also highlighted another theme, which we named *communication and empathy*. The theme captured students’ reflections on the importance of nuanced communication, patient perspectives, and emotional responses to cancer. Several students linked these insights to their future role as physicians, emphasizing the need to adapt communication to individual patients rather than relying on generalized assumptions about cancer: *“I have reflected on the importance of tailoring communication to each individual patient.”*

Finally, a theme emerged viewing *cancer as part of life*, with reflections like *“Perhaps we should focus on reconciling ourselves with the idea that cancer will affect most of us*,*”* and *“I have reflected on what it is like to live with cancer*,* how healthcare professionals meet patients with cancer*,* the strengths and weaknesses of today’s cancer care*,* and existential questions linked to being human.”* Compared with the pre-course associations dominated by *fear and uncertainty*, these reflections suggested a more accepting and contextualized understanding of cancer as a common human experience.

Overall, the students came to the course with a predominantly fatalistic and daunting view, focused on cancer’s threatening nature and the physical burdens of treatment, whereas their end-of-course reflections suggested a shift towards a broadened perspective. Many students emphasized the importance of examining biases in how cancer is represented in the media, acknowledging the responsibility of healthcare professionals to foster informed discussions. They articulated a nuanced appreciation for cancer’s multifaceted nature, recognizing it as more than a death sentence and acknowledging personal experiences, survivability, and acceptance as part of a broader process of understanding. These reflections suggested that students had begun to connect their broadened understanding of cancer with their future professional role, particularly through greater attention to communication, empathy, and the impact of language in patient interactions.

## Discussion

In this study, we evaluated an integrative learning design that used framing analysis as an educational tool to support critical and integrative thinking among medical students, specifically focusing on the topic of cancer. This learning process was assessed through pre-course free associations and post-course reflections that explored the students’ attitudes and understanding of the disease. Notably, the pre- and post-questionnaires differed: the first asked for the students’ free associations about cancer, while the second solicited more specific reflections on learning outcomes. Comparisons of the two responses should therefore be interpreted with caution. Nonetheless, the observed differences suggested meaningful changes in students’ perceptions and engagement with cancer-related content.

As a general methodological consideration, it is also important to note that this type of educational research conducted by the course designers and instructors may introduce researcher bias. Our dual roles as both educators and researchers might have influenced the description of course design, data interpretation, and presentation of the findings. However, in alignment with the project’s overarching goal of promoting critical thinking, we have sought to critically evaluate the results and present our work in a transparent and systematic manner.

Addressing our first research question, the students appeared to develop a working understanding of framing analysis, applying it to news articles, social media, and their own free associations about cancer. Furthermore, they used both inductive and deductive framing techniques to explore their assigned research questions, demonstrating that they were able to apply the concept beyond the initial workshop exercises. These findings support the educational utility of implementing a clearly defined framing scheme, such as the one developed by Murray et al. [2], as an effective method for stimulating critical reading and discussion. In alignment with the learning design previously presented by Carver et al. [16], our study demonstrates the practical feasibility of using framing analysis to support students’ analytical and reflective learning, thereby extending this method into medical education research and the field of cancer education.

Addressing our second research question, the thematic analysis of the students’ pre-course associations and post-course reflections suggested meaningful changes in their understanding of cancer. While initial responses reflected emotionally charged and often stereotypical associations with cancer, the end-of-course reflections indicated a shift toward a broader perspective, critical engagement with diverse sources of information, and greater recognition of the complex role of cancer in society. These changes were evident in the written reflections and were consistent with students’ application of framing analysis in their final team presentations, highlighting analytical engagement, integration of multiple perspectives, and an appreciation of the multifaceted nature of cancer.

Viewed in the broader context of medical education practice and research, this study highlights the potential value of an interdisciplinary learning design that addresses cancer—typically regarded as a biomedical phenomenon—through the lens of communication studies. The emphasis on critical thinking and analytical skills addresses a growing priority in medical education to equip students with the ability to evaluate and analyze diverse forms of information related to complex phenomena such as cancer [4, 11].

By employing framing analysis as an educational method, our study aligns with constructivist principles that actively engage students in the learning process and the creation of meaning [23]. It also underscores the importance of media literacy in developing students’ abilities to critically appraise health information and public narratives about disease [24]. Overall, these findings illustrate how qualitative, interdisciplinary approaches may broaden students’ understanding of complex health topics and foster critical, reflective thinking with relevance to patient care and public health contexts.

These results should be interpreted in light of the course’s elective format, small-group design, and specific institutional context. An interactive and collaborative course like this relies heavily on “the human factor,” particularly the engagement and enthusiasm of the instructors, as well as close supervision and collaboration with external partner organizations. The course is resource-intensive and may not be directly transferable to other medical programs without contextual adaptation. Transitioning from an elective to integration into the general curriculum may require a simplified version, while retaining the core pedagogical sequence of introduction to framing, guided application of a framing scheme, and independent use of framing analysis in student projects.

The core concept, as well as the more specific approach, may also be adaptable to other areas of medical education. Integrating framing analysis with related subjects such as clinical communication, community health, and medical ethics could facilitate broader application. The concept may also be adapted to other clinical topics, for example by applying research on the media framing of diabetes in a manner similar to how we applied framing analysis to cancer [25]. Furthermore, the approach could be relevant for target groups within and outside higher education, including healthcare professionals, health advocates, and health journalists.

While aggregate student preference data suggest that the course attracted substantial interest, the gender imbalance indicated that this interest was not evenly distributed across student groups. Across three course iterations, male students were markedly underrepresented among enrolled students, suggesting that they were less likely to select this type of interdisciplinary and qualitative approach to medical education. Although we do not have data on why students selected or did not select this particular elective, the gender imbalance may reflect broader gendered patterns in medical education. Previous research suggests that female students tend to show stronger patient-centered attitudes, which aligns with the course’s emphasis on communicative and relational aspects of medicine [26].

Future research should explore the applicability of framing analysis across diverse medical education contexts, including its long-term impact on students’ professional development, clinical reasoning, and patient communication. In addition, further work should examine how qualitative and communication-based methods can be integrated with biomedical content to foster reflective, interdisciplinary thinking and help healthcare professionals navigate complex clinical and societal challenges.

In conclusion, this study demonstrates the educational value of framing analysis as a structured pedagogical tool for exploring complex health phenomena in medical education. Applied to cancer communication, the approach helped students move beyond familiar biomedical and fatalistic understandings of cancer and engage more critically with the social, cultural, communicative, and existential dimensions of disease. By combining communication, media literacy, qualitative analysis, teamwork, and reflection, framing analysis offers a promising approach for strengthening interdisciplinary and reflective learning in health professions education.

## Supplementary Information

Supplementary Material 1.

Supplementary Material 2.

## Data Availability

Additional anonymized materials are available from the corresponding author on request.
