# Supplementary material for: Framing analysis as a pedagogical tool for broadening medical students’ understanding of cancer: a course evaluation
Source: BMC Med Educ. 2026 Jul 3;26:1513. doi: 10.1186/s12909-026-09851-y (PMC13602542; doi:10.1186/s12909-026-09851-y)
Supplement: Supplementary file 1 [file 12909_2026_9851_MOESM1_ESM.docx]

**Evalueringskriterier MED3056:**

|  | **Ja** | **Delvis** | **Nei** |
| --- | --- | --- | --- |
| Presenterer bakgrunnen for problemstillingen. |  |  |  |
| Forklarer hvorfor studien er viktig og interessant. |  |  |  |
| Angir hva vet vi om problemstillingen fra før (fra forskningslitteraturen) |  |  |  |
| Angir hva vi ikke vet – knowledge gap. |  |  |  |
| Forklarer hvilke materialer og metoder de har benyttet og hvorfor. |  |  |  |
| Presenterer resultatene på en tydelig og systematisk måte og understreker hvordan de besvarer forskningsspørsmålet. |  |  |  |
| Kritisk diskuterer resultatene. |  |  |  |
| Presenterer styrker og svakheter ved analysen (f.eks. generaliserbarhet og validitet)? |  |  |  |
| Diskuterer hvordan resultatene passer med det som er vist fra før (eksisterende kunnskap)? |  |  |  |
| Diskuterer praktiske implikasjoner av funnene. Hva kan de brukes til? |  |  |  |
| Presenterer en tydelig konklusjon – hva er «the take home message» (2-4 punkter). |  |  |  |
| Formidler tydelige indikasjoner på godt teamarbeid. Alle er med, god kommunikasjon. |  |  |  |
| Legger ved en oversiktlig kildehenvisning / referanseliste. |  |  |  |

**Evaluation Criteria MED3056:**

|  | **Yes** | **Partly** | **No** |
| --- | --- | --- | --- |
| Presents the background for the research question. |  |  |  |
| Explains why the study is important and interesting. |  |  |  |
| Describes what is already known about the research question based on the research literature. |  |  |  |
| Identifies what is not yet known—the knowledge gap. |  |  |  |
| Presents the results in a clear and systematic manner and emphasizes how they answer the research question. |  |  |  |
| Presenterer resultatene på en tydelig og systematisk måte og understreker hvordan de besvarer forskningsspørsmålet. |  |  |  |
| Critically discusses the results. |  |  |  |
| Presents the strengths and weaknesses of the analysis, for example generalizability and validity. |  |  |  |
| Discusses how the results fit with previous findings and existing knowledge. |  |  |  |
| Discusses practical implications of the findings: What can they be used for? |  |  |  |
| Presents a clear conclusion—the “take-home message” in 2–4 points. |  |  |  |
| Shows clear indications of good teamwork: all team members participate and communication is effective. |  |  |  |
| Includes a clear source list or reference list. |  |  |  |
