# Supplementary material for: Framing analysis as a pedagogical tool for broadening medical students’ understanding of cancer: a course evaluation
Source: BMC Med Educ. 2026 Jul 3;26:1513. doi: 10.1186/s12909-026-09851-y (PMC13602542; doi:10.1186/s12909-026-09851-y)
Supplement: Supplementary file 2 [file 12909_2026_9851_MOESM2_ESM.docx]

**Supplementary information**

Transforming Medical Students’ Understanding of Cancer Through Framing Analysis

Breivik *et al.*

**Questionnaire – Post-test**

**Q1**

*English:* What is the most important thing you learned in this course?

*Norwegian:* Hva vil du si er det viktigste du har lært i kurset?

**Q2**

*English:* Explain how the course has influenced your view of cancer?

*Norwegian:* Forklar hvordan kurset har påvirket synet ditt på kreft?

**Q3**

*English:* How do you think what you have learned might influence you in your further studies and medical practice?

*Norwegian:* Hvordan tror du det du har lært kan påvirke deg videre i studiet og legeyrket?
